# Glomerular hyperfiltration as a therapeutic target for CKD

**DOI:** 10.1093/ndt/gfae027

**Published:** 2024-02-02

**Authors:** Mehmet Kanbay, Sidar Copur, Cicek N Bakir, Adrian Covic, Alberto Ortiz, Katherine R Tuttle

**Affiliations:** Department of Medicine, Division of Nephrology, Koc University School of Medicine, Istanbul, Turkey; Department of Medicine, Koc University School of Medicine, Istanbul, Turkey; Department of Medicine, Koc University School of Medicine, Istanbul, Turkey; Nephrology Clinic, Dialysis and Renal Transplant Center – ‘C.I. Parhon’ University Hospital and ‘Grigore T. Popa’ University of Medicine, Iasi, Romania; Department of Nephrology and Hypertension, IIS-Fundación Jiménez Díaz UAM, Madrid, Spain; Division of Nephrology, University of Washington, Seattle, WA, USA; Providence Medical Research Center, Providence Health Care, WA, USA

**Keywords:** angiotensin, chronic kidney disease, glomerular haemodynamics, glomerular hyperfiltration, sodium–glucose co-transporter 2 inhibitors

## Abstract

The global burden of chronic kidney disease (CKD) is high and increasing. Early diagnosis and intervention are key to improve outcomes. Single-nephron glomerular hyperfiltration is an early pathophysiologic manifestation of CKD that may result in absolute glomerular hyperfiltration, i.e. a high glomerular filtration rate (GFR), or be associated with normal or low GFR because of nephron loss (relative glomerular hyperfiltration). Even though compensatory glomerular hyperfiltration may contribute to maintain kidney function after the loss of kidney mass, the associated increased glomerular capillary pressure and glomerular and podocyte size drive podocyte loss, albuminuria and proximal tubular overload, contributing to CKD progression. In this regard, all kidney protective drugs in clinical use so far, from renin–angiotensin system blockers to mineralocorticoid receptor blockers to sodium–glucose co-transporter 2 inhibitors to tolvaptan, induce an early dip in glomerular filtration that is thought to represent reversal of hyperfiltration. As glomerular hyperfiltration may be present early in the course of kidney disease, its recognition may provide an effective intervention window that may predate current criteria based on high albuminuria or loss of GFR. Nevertheless, there is no diagnostic method with high sensitivity and specificity to identify single-nephron glomerular hyperfiltration, except when it leads to obvious absolute glomerular hyperfiltration, as observed in the early stages of diabetic kidney disease when nephron mass is still preserved. We now review the concept of glomerular hyperfiltration as an indicator of CKD risk, including definitions, challenges in diagnosis and evaluation, underlying pathophysiological mechanisms, potential therapeutic approaches and unanswered questions.

## INTRODUCTION

Around 850 million people have chronic kidney disease (CKD) worldwide and the health, economic and environmental burden of CKD is increasing [1]. The impact of treatment for CKD [e.g. renin–angiotensin system (RAS) inhibitors, sodium–glucose co-transporter 2 (SGLT2) inhibitors] is limited by late prescription; they are used to treat CKD, but current guidelines define CKD as the presence of markers of kidney damage, such as albuminuria ≥30 mg/day (i.e. ≈10-fold above physiological levels), or estimated glomerular filtration rate (eGFR) <60 ml/min/1.7  m^2^ (i.e. 50% lower than physiological levels) for ≥3 months. A potentially long pre-CKD period is often unrecognized and untreated. Therefore, there is a need for earlier detection of high-risk patients. All current nephroprotective medications cause an early dip in eGFR, which is thought to represent decreased glomerular hyperfiltration. In this regard, decreasing glomerular hyperfiltration may be considered a therapeutic target. We now discuss the concept of glomerular hyperfiltration as an indicator of CKD risk, including definitions, challenges in diagnosis and evaluation, underlying pathophysiological mechanisms, potential therapeutic approaches and unanswered questions.

### Absolute and relative glomerular hyperfiltration

Glomerular hyperfiltration was first identified by Brenner in 1983 in a case of antepartum haemorrhage requiring multiple blood product transfusions due to abruption of the placenta that lead to acute kidney injury with a haemodialysis requirement during the long-term follow-up period [2]. Although glomerular hyperfiltration theory gained considerable attention and was considered a potential therapeutic target in kidney diseases in the 1990s [3], scientific interest decreased in the early 2000s that may be attributable to difficulties in detection of clinical or laboratory indicators, histopathological findings and the scarcity of therapeutic options. Identification of glomerular hyperfiltration is challenging due to methodological limitations and physiological variability (Table 1). Glomerular hyperfiltration has been defined as a GFR higher than 2 standard deviations (SDs) above mean values for age and gender [4, 5], however, absolute GFR results from the sum of all the single nephron glomerular filtration rates (snGFRs). Thus absolute GFR may be normal or even low despite relative glomerular hyperfiltration when the snGFR is increased in remaining nephrons after the loss of nephron mass, a common occurrence in persons with CKD.

**Table 1:** Absolute and relative glomerular hyperfiltration.

Absolute glomerular hyperfiltration: eGFR >2 SD above the expected GFR for age and gender.
Absolute GFR = single-nephron filtration rate × total number of functioning nephrons
Methods for GFR assessment in the clinic: eGFR from serum creatinine or cystatin C Creatinine clearance Iohexol ^99^Tc-DTPA (diethylenetriamine pentaacetate)
Methods for nephron number assessment:
Total nephron number = nephron density on biopsy specimen × total volume
Total volume assessment tools: Computed tomography Magnetic resonance imaging
Relative glomerular hyperfiltration: eGFR within reference range with an elevated single-nephron filtration rate and a reduced number of functioning nephrons
Pragmatic approach in the clinic (hypothetical): eGFR decrease within a certain (to be defined) range in response to kidney protective drug(s) (to be defined) at a certain timepoint (to be defined)

GFR is usually estimated from serum creatinine or cystatin C. Thus non-GFR determinants of lower serum creatinine (e.g. low muscle mass in malignancy) or of cystatin C values may cause eGFR values suggestive of glomerular hyperfiltration. Conversely, interference with tubular secretion of creatinine or high muscle mass may increase serum creatinine levels, hiding glomerular hyperfiltration. Absolute glomerular hyperfiltration can only be diagnosed or confirmed by measured GFR (mGFR), although it is still very challenging, as it may be over- or underestimated by serum creatinine or cystatin C–based estimations [6, 7].

Hypothetically, mean snGFR may be calculated by dividing the total mGFR by the total number of nephrons, if both could be accurately determined, but this would still not account for nephron heterogeneity. Recent approaches have measured GFR and estimated nephron number from nephron density on biopsy specimens and total cortical kidney volume via imaging (e.g. computed tomography, magnetic resonance imaging) [8]. These calculations assume a homogeneous distribution of healthy glomeruli across the cortex and homogeneity in glomerular size and snGFR, assumptions that may not be fulfilled in individuals with or at risk of CKD, which would be the context of use.

A pragmatic approach may explore whether initiation of kidney protective drugs results in a dip in eGFR. Observation of a dip in eGFR would be consistent with the existence of increased snGFR, independent of the baseline eGFR. While post hoc analysis of randomized controlled trials (RCTs) have described an association of the early dip in eGFR with long-term stabilization of kidney function [9], we are not aware of prospective studies that have assessed the existence and magnitude of the early dip in eGFR as a measure of single nephron glomerular hyperfiltration or a tool to initiate therapy aimed at long-term kidney protection. Moreover, such clinical outcomes should be carefully assessed, as GFR estimations based on serum creatinine or cystatin C have considerable limitations that may over- or underestimate actual GFR [10]. Additionally, the optimal drug or drug combinations to be used, units to express the magnitude of the eGFR decrease (ml/min/1.73 m^2^, percent decrease in eGFR), optimal timing for such tests and range of expected results by age, sex or baseline eGFR should be defined.

### Physiological and pathological glomerular hyperfiltration

Glomerular filtration is dynamic, allowing for adaptation to physiological needs. In a classic example, a high-protein meal is associated to transient glomerular hyperfiltration. Pregnancy also exemplifies physiological glomerular hyperfiltration. Pregnancy causes systemic and local haemodynamic changes, including increased cardiac output, vasodilatation and extracellular and intracellular volume expansion mediated via renal salt and water retention [11]. In the first and second trimesters, renal plasma flow and GFR increase by 40–60%, while in the third trimester renal plasma flow declines but GFR remains stable, reflecting an elevated filtration fraction. Thus glomerular hyperfiltration results from a 50% increase in filtration fraction and a 16% increase in glomerular capillary hydrostatic pressure [12]. Potential underlying mechanisms include blunted response to angiotensin II, elevated prostacyclin and progesterone levels and, possibly, placental factors (e.g. relaxin). Glomerular hyperfiltration is rapidly reversible following pregnancy and does not increase the risk of CKD, although it may accelerate CKD in women with pre-existing CKD [13].

Increased snGFR in CKD is initially adaptative and allows maintaining GFR despite nephron loss, but, as discussed below, eventually becomes a pathological phenomenon that contributes to CKD progression. Other conditions in which glomerular hyperfiltration may be pathological include diabetes mellitus (DM), obesity, malignancies, sleep apnoea and high altitude [4, 5].

### Pathophysiology of glomerular hyperfiltration

**Figure 1:**
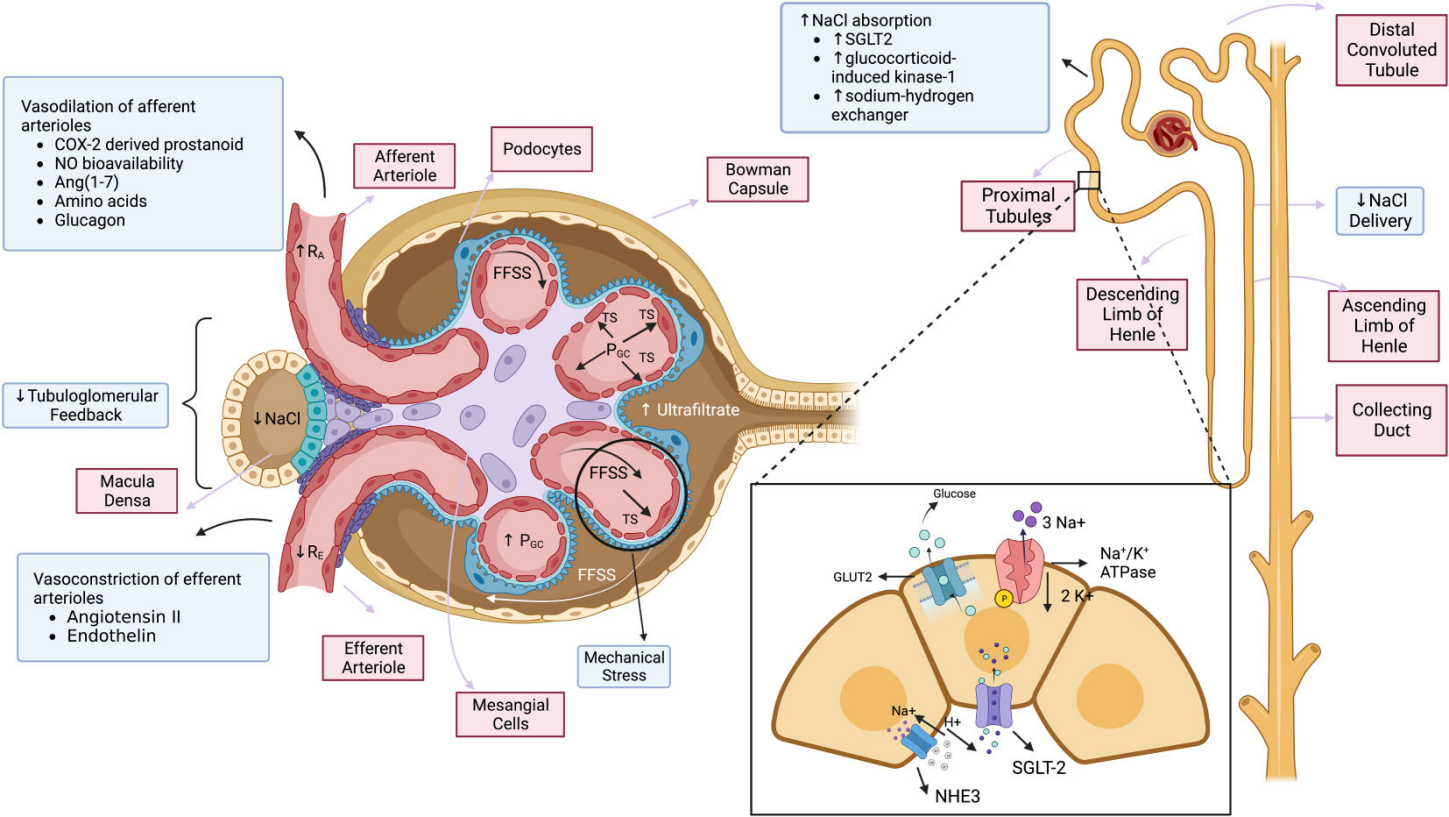
Tubular and vascular mechanisms of glomerular hyperfiltration. The net effect of vascular mediators generates increased glomerular filtration. Diminished sodium chloride delivery to the macula densa results in deactivation of tubuloglomerular feedback and vasodilation of efferent arterioles. Shear stress results from increased blood and ultrafiltrate flow and is parallel to the surface. Tensile stress arises from increased hydrostatic pressure perpendicular to the capillary wall. FFSS: fluid flow shear stress; PGC: glomerular capillary pressure; RA: resistance of the afferent arteriole; RE: resistance of the efferent arteriole; TS: tensile stress.

Glomerular hyperfiltration is a complex pathophysiological event, best characterized in patients with DM or obesity. Drivers and consequences of glomerular hyperfiltration have been identified (Fig. 1) [4, 5].

#### Drivers of glomerular hyperfiltration

Drivers of glomerular hyperfiltration include increased glomerular capillary pressure and glomerular size and loss of tubuloglomerular feedback.

Vasodilation of afferent arterioles and/or vasoconstriction of efferent arterioles increase the glomerular capillary hydrostatic pressure, causing glomerular hyperfiltration. Angiotensin II induces vasoconstriction of efferent arterioles, while angiotensin [1–7], nitric oxide and endothelial cyclooxygenase-2 (COX-2)-derived prostanoids cause vasodilation of afferent arterioles and vasopressin V2 receptors cause vasoconstriction of afferent arterioles [5]. In contrast, endothelin-1 causes vasoconstriction of both afferent and efferent arterioles, and systemic endothelin-1 infusion acutely decreased renal blood flow, GFR and urinary sodium excretion in healthy human volunteers, an effect prevented by atrasentan, an endothelin receptor A blocker [14].

Remnant nephrons increase in size, including increased glomerular size, which would increase the filtration surface and filtration fraction should glomerular pressure remain stable. Podocytes do not proliferate and also increase in size, thus becoming more exposed to increased glomerular capillary hydrostatic pressure.

Increased sodium chloride availability at the macula densa causes afferent arteriolar vasoconstriction (tubuloglomerular feedback), decreasing GFR and distal sodium delivery. Excess glomerular filtration of glucose in DM will increase SGLT2 expression and activity, resulting in higher proximal tubular sodium and chloride reabsorption and fewer solutes delivered to the macula densa, thus decreasing the tubuloglomerular feedback, leading to afferent arteriolar vasodilation and glomerular hyperfiltration [15]. Increased proximal tubular sodium reabsorption in obese subjects is thought to depend on compression of kidney parenchyma by visceral and perirenal adipose tissue, thus increasing RAS and sympathetic nervous system activity [16], although glycaemia correlates with GFR even within the subdiabetic range.

#### Consequences of glomerular hyperfiltration

Glomerular hyperfiltration contributes to CKD progression through mechanical stress, leading to podocyte loss, albuminuria and tubular injury, as well as tubular work overload.

##### Mechanical stress

**Figure 2:**
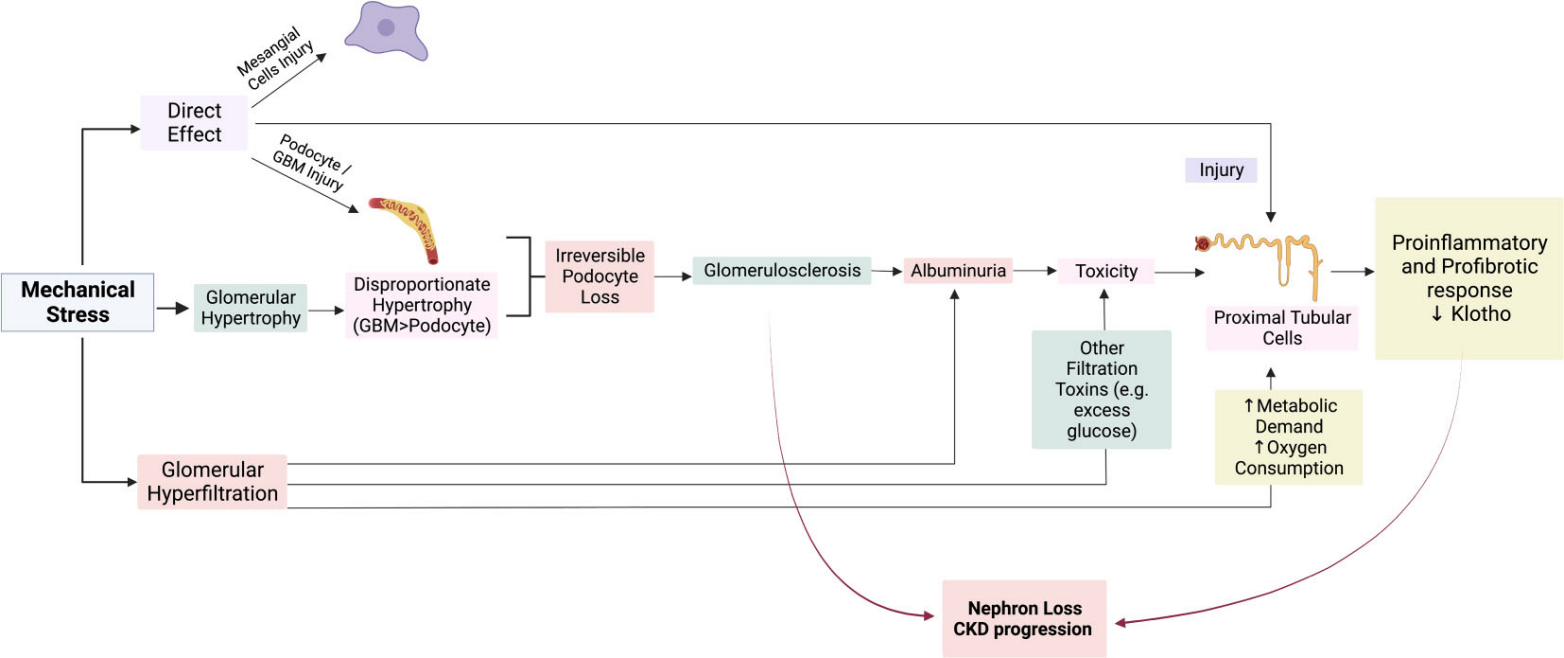
The pathological effects of mechanical stress on nephrons simplified in a schema. Mechanical stress induces a direct injury in podocytes, GBM, mesangial cells and proximal tubule cells. Simultaneously, mechanical stress induces glomerular hypertrophy and causes disproportionate hypertrophy in podocytes and the GBM. Direct injury and disproportionate hypertrophy result in irreversible podocyte loss and glomerulosclerosis. This structural damage, coupled with glomerulosclerosis and increased metabolic demand due to hyperfiltration, gives rise to observable clinical manifestations such as albuminuria, contributing to their heightened toxicity. Increased metabolic demand, toxicity and direct injury result in pro-inflammatory and profibrotic response coupled with decreased klotho, an anti-aging protein crucial for renal health, and causes nephron loss and CKD progression.

Mechanical stress may negatively impact the filtration barrier and tubular cells. Increased intraglomerular hydrostatic pressures and increased ultrafiltrate flow cause mechanical tensile and shear stress (Fig. 2) [17]. Blood exerts tensile stress perpendicular to the capillary walls [endothelial cells, glomerular basement membrane (GBM), podocytes]. The magnitude of tensile stress depends on pressure, capillary wall radius and wall thickness. Shear stress is applied parallel to the surface of cells. Endothelial cells experience shear stress due to blood flow in capillaries, while podocytes and tubular cells encounter shear stress from ultrafiltrate flow. The magnitude of shear stress depends on fluid viscosity, flow velocity and the size of the ultrafiltrate column.

Increased tensile stress by glomerular hyperfiltration results in GBM expansion and triggers an adaptive response in podocytes, resulting in glomerular and podocyte hypertrophy and injury [18]. Hypertrophy occurs shortly after the onset of DM. Podocyte hypertrophy results in stretching and thinning of podocyte bodies, contributing to podocyte foot effacement and detachment and loss of the podocytes. Loss of podocytes brings denudation of the GBM, adherence of the capillary wall to Bowman's capsule, synechia and eventually the onset of segmental sclerosis [18]. Some mediators, such as angiotensin II also contribute to podocyte hypertrophy and elicit a stress response characterized by release of pro-inflammatory and profibrotic mediators [19]. Podocyte injury causes albuminuria and, given their limited capacity for regeneration, podocyte loss contributes to irreversible glomerular injury. Mechanical stress also acts on mesangial cells [20]. Finally, the increased ultrafiltrate volume and expanded tubular space creates shear and tensile stress in proximal tubules, resulting in hypertrophy and proliferation of tubular cells and increased expression of the luminal sodium–hydrogen antiporter 3 (NHE3) transporter and the basolateral Na^+^/3HCO_3_^−^ co-transporter [21], increasing sodium reabsorption, decreasing delivery of sodium to the macula densa and deactivating tubuloglomerular feedback and further increasing glomerular hyperfiltration [17].

##### Tubular overload

Glomerular hyperfiltration creates a vicious cycle, elevating metabolic demand and oxygen consumption by proximal tubules that should reabsorb approximately two-thirds of the glomerular ultrafiltrate. In the presence of CKD, this may occur under conditions of limited oxygen availability, due to capillary rarefaction and, as interstitial inflammation and fibrosis progress, an adverse microenvironment. On top of that, the additional amount of potential proximal tubular toxins that are filtered in excess due to glomerular hyperfiltration may further damage proximal tubules. Prominent among them is albumin, filtered in excess because of the combination of hyperfiltration (although <1% of albumin is filtered by normal glomeruli, this may amount to up to 3 g/day) and podocyte injury. Additional filtered toxins may be environmental or endogenous. In haematuric nephropathies or anticoagulated patients, larger glomeruli may leak increased numbers of red blood cells, some of which may release their contents early on, leading to haem toxicity and iron overload in proximal tubules. Iron overload predisposes to ferroptotic cell death, a key regulated necrosis pathway during kidney injury [22].

##### Glomerular hypertrophy

As glomerular hypertrophy is involved in the pathogenesis of various kidney diseases, including diabetes mellitus, obesity-associated kidney disease and focal segmental glomerulosclerosis, such a pathological event is the initial histopathological alteration in response to glomerular hyperfiltration [23–25]. Even though albuminuria/proteinuria and a decrease in eGFR are commonly used clinical parameters in the evaluation of CKD progression, glomerular hypertrophy precedes the development of such clinical parameters and also represents a superior indicator for CKD progression [24, 26]. Even though the clinical significance of glomerular hypertrophy is well-established, current pathological classifications rarely rely on renal corpuscle or glomeruli size [23]. Moreover, assessment of glomerular size is often challenging due to significant heterogeneity between glomeruli from a single kidney, the presence of sclerotic or collapsed glomeruli in biopsy specimens, sample size, the method of measurement and differentiation of physiologically enlarged glomeruli from a ‘diseased’ state [27]. There is currently no gold standard definition for pathological glomeruli size that effectively distinguishes pathological state from physiological state. Nevertheless, renal corpuscle enlargement >1.5 times its physiological size has been proposed as a criterion for the pathological state, based on a study of immunoglobulin A nephropathy (IgAN) [28]. In most cases, injured glomeruli increase in size before sclerosing or collapsing, which may lead to difficulty in the interpretation of results [29]. Thus the mean diameter or SD of glomeruli size may not actually reflect the actual disease state. To conclude, glomerular hypertrophy is a key and early pathological event with well-established correlation with disease progression despite the lack of clear and effective methods for analysis in histopathological specimens. Moreover, there is no clinical or laboratory assessment tool that may predict such a hypertrophic state except for invasive histopathological specimens. There is a clear need for future large-scale pre-clinical and clinical studies investigating glomerular hypertrophy assessment methods with high sensitivity and specificity.

### Glomerular hyperfiltration and kidney disease

The initial stage of diabetic kidney disease consists of glomerular hyperfiltration in both type 1 and type 2 DM. As predicted by the negative consequences of hyperfiltration discussed above, glomerular hyperfiltration is a risk factor for future albuminuria independent of metabolic control and of rapid eGFR loss [30]. Indeed, among 1 024 977 participants, supraphysiological eGFR, as well as low eGFR, high albuminuria and the presence of DM were risk factors for kidney failure and death over 8.5 years of follow-up [31]. Similar observations have been made for hypertension. Persons with essential hypertension and higher baseline eGFR were at higher risk of increased creatinine levels at 6 years of follow-up [32] and had a larger increase in albuminuria at 7.8 years [33]. eGFR also predicted microalbuminuria at 3.1 years in stage 1 hypertension patients in adjusted analysis [34]. Glomerular hyperfiltration was also associated with the development of CKD in the general population [35].

#### Therapeutic perspective

All kidney protective drugs in clinical use so far, and some drugs in clinical development that have shown kidney protective potential, induced an early decrease in GFR, although this should be evaluated with caution due to the limitation of GFR estimation tools (Table 2), that is thought to represent a reversal of hyperfiltration, further supporting a causal link between hyperfiltration and CKD progression and the concept that glomerular hyperfiltration itself is a bona fide therapeutic target. Evidence for kidney protection by the drugs discussed next has been convincingly demonstrated in patients with CKD in whom RCTs were performed with a primary endpoint of kidney protection. Additionally, for SGLT2 inhibitors and incretin-based therapies, evidence emerging from the post hoc analysis of secondary endpoints in patients with type 2 DM without baseline CKD further supports the concept that the kidney benefits of targeting glomerular hyperfiltration may extend to people without CKD but at high risk of CKD, i.e. they support the concept that glomerular hyperfiltration may be a therapeutic target in the prevention of CKD [36].

**Table 2:** Early decrease in eGFR in clinical trials investigating the effects of kidney protective medications.

Family	Drug	Reference	Subjects	Timepoint	Baseline eGFR or serum creatinine	eGFR decrease versus baseline
RAS inhibitors	Losartan 50–100 mg/day	RENAAL trial [38]	Adults with serum creatinine of 1.3–3.0 mg/dl and UACR >300 mg/g	Mean 3.4 years	1.9 mg/dl for losartan group 1.9 mg/dl for placebo group	−2.3 ml/min/1.73 m^2^ at month 3 for losartan group −1.6 ml/min/1.73 m^2^ at month 3 for placebo group (*P*-value = .031)
Aldosterone/MR inhibitor	Finerenone 20 mg/day plus dapagliflozin 10 mg/day + RAS blockers	[41]	Adults with eGFR of 25–45 ml/min/1.73 m^2^ and UACR 150–2000 mg/g	8 weeks	37 ml/min/1.73 m^2^ for patients initiated with finerenone 30 ml/min/1.73 m^2^ for patients initiated with dapagliflozin	−7 ml/min/1.73 m^2^ at week 8 in both groups
SGLT2 inhibitor	Empagliflozin 10 mg/day + RAS blockers	[43]	Adults with eGFR of 20–90 ml/min/1.73 m^2^ and UACR >200 mg/g	Median 2 years	37.4 ml/min/1.73 m^2^ for empagliflozin group 37.3 ml/min/1.73 m^2^ for placebo group	eGFR decrease total slope of −2.16 and chronic slope of −1.37 for empagliflozin group eGFR decrease total slope of −2.92 and chronic slope of −2.75 for placebo group
Endothelin-A receptor antagonists	Zibotentan 0.25 or 1.5 mg/day + dapagliflozin 10 mg + RAS blockers	ZENITH-CKD [55]	Adults with eGFR >20 ml/min/1.73 m^2^ and UACR of 150–5000 mg/g	12 weeks	45.2 ml/min/1.73 m^2^ for dapagliflozin plus placebo 47.4 ml/min/1.73 m^2^ for dapagliflozin plus zibotentan 1.5 mg/day 48.4 ml/min/1.73 m^2^ for dapagliflozin plus zibotentan 0.25 mg/day	−0.8 ml/min/1.73 m^2^ at week 1 and −1.1 ml/min/1.73 m^2^ at week 12 for dapagliflozin plus zibotentan 1.5 mg compared with placebo 1.1 ml/min/1.73 m^2^ at week 1 and −1.2 ml/min/1.73 m^2^ at week 12 for dapagliflozin plus zibotentan 0.5 mg/day compared with placebo
V2R antagonist	Tolvaptan 45/15 or 60/30 or 90/30 mg/day	TEMPO 4:4 trial [59]	Adults with ADPKD with eGFR >30 ml/min/1.73 m^2^	24 months	72.3 ml/min/1.73 m^2^	−8.1 ml/min/1.73 m^2^ for early-treated group −10.6 ml/min/1.73 m^2^ for late-treated group

##### RAS inhibitors

An initial decrease in eGFR after RAS inhibition is common [37]. In the Reduction of Endpoints in NIDDM with the Angiotensin II Antagonist Losartan (RENAAL) clinical trial, losartan prevented CKD progression compared with placebo over 3.4 years in patients with type 2 DM and proteinuric CKD. A post hoc analysis revealed that the initial decrease in eGFR in the first 3 months with losartan treatment predicted slower CKD progression [38].

##### Mineralocorticoid receptor (MR) antagonists/aldosterone inhibitors

MR antagonists are kidney protective agents. In the Eplerenone Combination versus Conventional Agents to Lower Blood Pressure on Urinary Anti-albuminuric Treatment Effect (EVALUATE) study, eplerenone caused an initial decrease in eGFR and decreased albuminuria at 52 weeks in non-diabetic CKD patients with hypertension [39]. Finerenone, a selective non-steroidal MR antagonist, also caused an initial eGFR decrease and decreased CKD progression and improved cardiovascular outcomes in patients with type 2 DM and CKD [40]. Additionally, finerenone reduced mGFR at 8 weeks (−7 ml/min/1.73 m^2^; 95% CI −8 to −5) and albuminuria, both alone and when combined with dapagliflozin [41].

Aldosterone synthase inhibition is also being investigated to protect the kidneys and treat resistant hypertension. In a phase 2 RCT, BI 690517 caused an initial eGFR decrease and reduced albuminuria, with or without background SGLT2 inhibitor use, when added on top of RAS blockade in participants with CKD [eGFR 30–<90 ml/min/1.73 m^2^, urine albumin:creatinine ratio (UACR) ≥200 mg/g] [42].

##### SGLT2 inhibitors

SGLT2 inhibition decreases glomerular hyperfiltration through several mechanisms. It blocks proximal tubular sodium reabsorption and increases the sodium concentration delivered to the macula densa, restoring tubuloglomerular feedback [15]. Inhibiting sodium chloride reabsorption may also increase the intraluminal tubular pressure and, subsequently in the Bowman’s capsule, decrease filtration pressure [15].

Numerous clinical trials unequivocally demonstrate the kidney protective effects of SGLT2 inhibitors (canagliflozin, dapagliflozin, empagliflozin) in patients with CKD with or without type 2 DM and any degree of albuminuria [36, 43]. Moreover, post hoc analyses of cardiovascular outcome trials in >20 000 participants with type 2 DM who did not have CKD at baseline showed that diverse SGLT2 inhibitors prevent the development of CKD [36]. Overall, an initial GFR decrease in response to SGLT2 inhibition appears to reflect kidney protection by reducing glomerular hyperfiltration in the long term [9].

##### Incretin-based therapies

Glucagon-like peptide-1 receptor agonists (GLP-1RAs) are approved for treatment of type 2 DM and overweight/obesity and decrease albuminuria and improve kidney outcomes [44]. In a meta-analysis of eight RCTs, they decreased composite kidney outcomes by 21% in patients with type 2 DM {hazard ratio [HR] 0.79 [95% confidence interval (CI) 0.73–0.87]}, mainly because of decreased albuminuria. Additionally, positive results were communicated in a press release for the A Research Study to See How Semaglutide Works Compared to Placebo in People With Type 2 Diabetes and Chronic Kidney Disease (NCT03819153), which had a primary endpoint of persistent eGFR ≥50% from trial start, reaching kidney failure, death from kidney disease or death from cardiovascular disease. GLP-1 induced natriuresis and decreased eGFR at the end of a 3-hour infusion in insulin-resistant obese men with glomerular hyperfiltration [45], but no impact was observed in healthy men [46]. In an RCT of patients with type 2 DM, liraglutide decreased eGFR at the end of 1 year (100.6 ± 24.9 to 89.4 ± 25.7 ml/min/1.73 m^2^) [47]. The twincretin tirzepatide, an agonist of both the glucose-dependent insulinotropic polypeptide (GIP) and the GLP-1 receptors, caused an early decrease in eGFR in participants with type 2 DM and high cardiovascular risk when compared with insulin glargine, alone or combined with SGLT2 inhibitors, although such an early dip in eGFR should be carefully assessed [48].

GLP-1RAs inhibit several pathways involving glomerular hyperfiltration. Rat and human studies suggest that GLP-1 decreases NHE3-mediated bicarbonate reabsorption in proximal tubules, thereby augmenting natriuresis [49]. GLP-1 also increases urinary prostaglandin E2 excretion [50]. GLP-1RAs could also promote the release of vasoactive compounds such as nitric oxide and atrial natriuretic peptide and decreasing endothelin-1 [51].

##### Endothelin-A receptor antagonists (ERAs)

ERAs cause sodium retention that may trigger or aggravate heart failure and there is no pure ERA in clinical use to treat CKD [52]. The use of atrasentan has been linked to protection of renal function in a double-blind randomized placebo-controlled clinical trial conducted in 4711 adult patients with type 2 DM with an eGFR of 25–75 ml/min/1.73 m^2^, with fluid retention and anaemia as major adverse events [52]. In a prior phase 2 trial, no early (2 weeks) decrease in eGFR was observed [53]. However, a (non-statistically significant) progressive decrease of eGFR was observed up to week 12, which disappeared upon stopping the drug (Supplementary Fig. S1). The slow time course of eGFR decline was not consistent with an acute haemodynamic effect. Similarly, beneficial effects of ERA therapy on albuminuria and/or proteinuria have been illustrated in a few other clinical trials without demonstrable effects on eGFR [54]. As ERA therapy conveyed renal but not cardiovascular protection, combination therapies with SGLT2 inhibitors have been pursued, aimed at increasing kidney protection and ERA cardiovascular safety. The phase 2b double-blind randomized multicentred Zibotentan and Dapagliflozin for the Treatment of CKD (ZENITH-CKD) trial demonstrated the superiority of such combination therapy over SGLT2 inhibitor therapy alone in terms of albuminuria and systolic and/or diastolic blood pressure over 12 weeks [55]. Moreover, all treatment arms have led to an acute reduction in eGFR at week 1, with the largest decline in the combination treatment arm (zibotentan 1.5 mg plus dapagliflozin 10 mg), with a stable course during the clinical trial period. Two weeks following drug discontinuation in the trial, eGFR returned to baseline values in all treatment arms except the zibotentan 1.5 mg plus dapagliflozin group [55]. Ongoing pivotal phase 3 trials testing zibotentan plus dapagliflozin for patients with CKD and high proteinuria are expected to be completed by 2027 (NCT06087835). PROTECT (Efficacy and safety of sparsentan versus irbesartan in patients with IgA nephropathy) demonstrated that sparsentan, a dual endothelin angiotensin receptor antagonist, has better efficacy and similar safety than irbesartan in reducing proteinuria in IgAN [56], but sparsentan did not add an early eGFR decrease on top of that observed for the angiotensin receptor antagonist irbesartan [57]. In another randomized double-blind clinical trial involving systemic sclerosis patients, zibotentan increased eGFR over 52 weeks [58]. To conclude, the effects of ERA therapy on glomerular hyperfiltration or eGFR measurements are controversial, with limited available evidence in the literature despite the presence of consensus on their beneficial effects on proteinuria.

##### Tolvaptan

Tolvaptan is a vasopressin receptor 2 (V2R) antagonist in clinical use for autosomal dominant polycystic kidney disease (ADPKD). Tolvaptan was studied in ADPKD because of the role of V2R-mediated upregulation of cyclic adenosine monophosphate in cyst growth. Indeed, tolvaptan initially and over time decreases total kidney volume, reflecting decreased cyst size. Interestingly, tolvaptan also reversibly decreased eGFR, causing an early decrease, like other nephroprotective medications [59]. It is likely that this depends on afferent arteriolar vasoconstriction by blocking vasopressin V2R vasodilation [59]. Adding octreotide, a synthetic version of somatostatin, to tolvaptan further decreased eGFR and total kidney volume in the short term [60].

##### Acetazolamide

Acetazolamide is a carbonic anhydrase inhibitor that prevents sodium bicarbonate absorption in the proximal tubules, increasing sodium delivery to the macula densa and decreasing glomerular hyperfiltration via tubuloglomerular feedback, in healthy persons and those with DM or obese non-diabetics [61]. However, it induces proximal renal tubular acidosis, which may limit its long-term use and, contrary to the medications discussed above, has not been demonstrated to provide long-term kidney protection.

##### Weight loss

Weight loss after bariatric surgery or caloric restriction reduces glomerular hyperfiltration in obese people independent of type 2 DM [62]. Additionally, drugs such as GLP-1RAs also induce considerable weight loss [48].

### Unanswered questions

Despite evidence that glomerular hyperfiltration contributes to CKD progression and that kidney protective drugs decrease hyperfiltration, there are still unanswered questions that preclude considering hyperfiltration a bona fide therapeutic target in the treatment of CKD and, of even more interest, in the prevention of CKD (Table 3). On top of general questions regarding concepts, methods, interventions and outcomes, practical issues remain to be addressed resulting from the increasing number of kidney protective drugs that decrease glomerular hyperfiltration. These include defining resistance to kidney protection by certain drugs and clarification of additional effects on kidney protection by different medication families, and whether these may relate to resistance or additional effects on glomerular hyperfiltration. Moreover, there is a clear need for studies evaluating potential clinical indicators for glomerular hyperfiltration to identify such phenomenon as a valid therapeutic target. Some ongoing studies may start providing answers. An ongoing RCT (Adolescent Type 1 Diabetes Treatment With SGLT2i for hyperglycEMia & hyPerfilTration Trial; NCT04333823) is evaluating the effects of dapagliflozin in addition to insulin on glycaemic control and glomerular hyperfiltration over 16 weeks among adolescents with type 2 DM. Another (Effect of Farxiga on Renal Function and Size in Type 2 Diabetic Patients With Hyperfiltration; NCT02911792) is evaluating the effect of dapagliflozin and metformin on GFR among newly diagnosed hyperfiltrating adults with type 2 DM with or without A2 albuminuria.

**Table 3:** Knowns and unknowns about the concept of glomerular hyperfiltration.

Knowns	Unknowns
Concepts of absolute and relative glomerular hyperfiltration Haemodynamic and tubular theory for underlying pathophysiology Detrimental outcomes of glomerular hyperfiltration Potential therapeutic target as, so far, decreasing hyperfiltrationappearsto be a common feature of all approved kidney protectivedrugs	Concepts Definition of actionable hyperfiltration for prevention of CKD Definition of actionable hyperfiltration for treatment of CKD Definition of early responder/non-responder to specific therapeuticinterventions targeting glomerular hyperfiltration For above, define method of assessment, units of measurement,cut-off points (sex, age and baseline eGFR-adapted), predictionof response to preventive (before CKD develops) or therapeuticintervention (after CKD has developed), relationship of response tolong-term outcomes of kidney function and CKD complications Methods To identify patients with glomerular hyperfiltration among thosewith high eGFR To identify patients with relative glomerular hyperfiltration despitenormal or low eGFR For early identification of responders to different types ofinterventions Interventions Optimal targeted therapeutic approaches for glomerularhyperfiltration in diverse clinical situations Characterization of drug combinations that optimally decreaseglomerular hyperfiltration in diverse clinical situations Outcomes Characterization of long-term outcomes of targeting glomerularhyperfiltrationby diverse drugs or drug combinations indifferent clinical situations and for different purposes(prevention versus treatment of CKD)

## CONCLUSIONS

Glomerular hyperfiltration contributes to CKD progression and is decreased by all currently approved kidney protective drugs. Ongoing research into glomerular hyperfiltration holds promise for advancing early diagnostic and treatment approaches to CKD. In this regard, the development of convenient methods to assess the presence of single-nephron hyperfiltration may pave the way for the design of studies aimed at defining an earlier window of intervention to prevent rather than to treat CKD by prescribing a smart selection or combination of current or future nephroprotective drugs known to decrease hyperfiltration.

## Supplementary Material

gfae027_Supplemental_File

## Data Availability

All data are available in the article.
