# Supplementary figures and images for: Glomerular hyperfiltration as a therapeutic target for CKD
Source: Nephrol Dial Transplant. 2024 Feb 2;39(8):1228–38. doi: 10.1093/ndt/gfae027 (PMC12086678; doi:10.1093/ndt/gfae027)

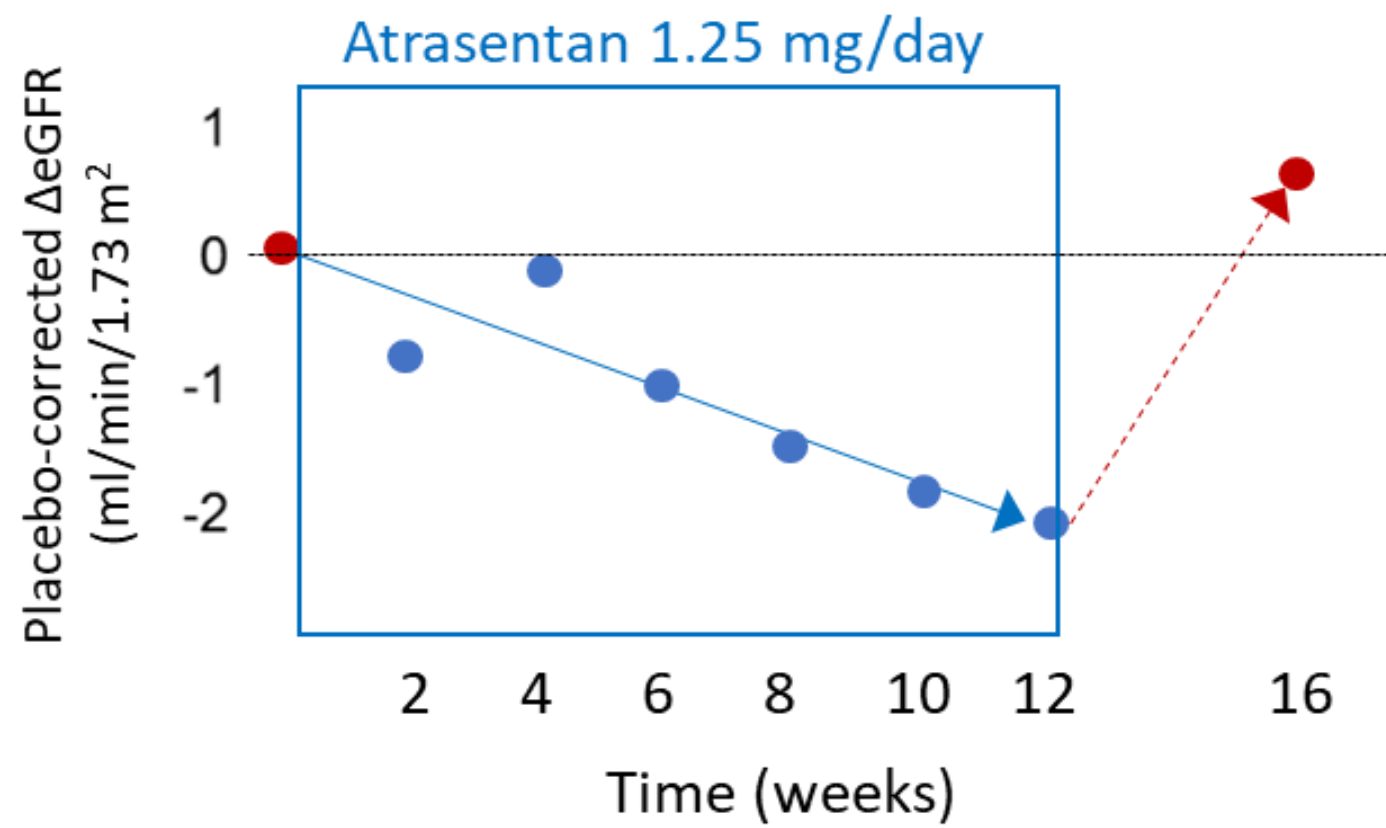

Supplement: gfae027_Supplemental_File [file gfae027_supplemental_file.pdf]
